# Dexamethasone Induced Osteocyte Apoptosis in Steroid‐Induced Femoral Head Osteonecrosis through ROS‐Mediated Oxidative Stress

**DOI:** 10.1111/os.14010

**Published:** 2024-02-21

**Authors:** Xinglong Zhang, Zhenhuan Yang, Qian Xu, Chunlei Xu, Wei Shi, Ran Pang, Kai Zhang, Xinyu Liang, Hui Li, Zhijun Li, Huafeng Zhang

**Affiliations:** ^1^ Department of Orthopaedics General Hospital of Tianjin Medical University Tianjin China; ^2^ Department of Orthopaedics Tianjin Nankai Hospital Tianjin China; ^3^ School of Integrative Medicine Tianjin University of Traditional Chinese Medicine Tianjin China

**Keywords:** apoptosis, osteocyte, oxidative stress, reactive oxygen species, steroid‐induced femoral head osteonecrosis

## Abstract

**Objective:**

Glucocorticoid (GC) overuse is strongly associated with steroid‐induced osteonecrosis of the femoral head (SINFH). However, the underlying mechanism of SINFH remains unclear. This study aims to investigate the effect of dexamethasone (Dex)‐induced oxidative stress on osteocyte apoptosis and the underlying mechanisms.

**Methods:**

Ten patients with SINFH and 10 patients with developmental dysplasia of the hips (DDH) were enrolled in our study. Sixty rats were randomly assigned to the Control, Dex, Dex + N‐Acetyl‐L‐cysteine (NAC), Dex + Dibenziodolium chloride (DPI), NAC, and DPI groups. Magnetic resonance imaging (MRI) was used to examine edema in the femoral head of rats. Histopathological staining was performed to assess osteonecrosis. Immunofluorescence staining with TUNEL and 8‐OHdG was conducted to evaluate osteocyte apoptosis and oxidative damage. Immunohistochemical staining was carried out to detect the expression of NOX1, NOX2, and NOX4. Viability and apoptosis of MLO‐Y4 cells were measured using the CCK‐8 assay and TUNEL staining. 8‐OHdG staining was conducted to detect oxidative stress. 2′,7′‐Dichlorodihydrofluorescein diacetate (DCFH‐DA) staining was performed to measure reactive oxygen species (ROS). The expression of NOX1, NOX2, and NOX4 in MLO‐Y4 cells was analyzed by Western blotting. Multiple comparisons were performed using one‐way analysis of variance (ANOVA).

**Results:**

In patients and the rat model, hematoxylin–eosin (HE) staining revealed a significantly higher rate of empty lacunae in the SINFH group than in the DDH group. Immunofluorescence staining indicated a significant increase in TUNEL‐positive cells and 8‐OHdG‐positive cells in the SINFH group compared to the DDH group. Immunohistochemical staining demonstrated a significant increase in the expression of NOX1, NOX2, and NOX4 proteins in SINFH patients compared to DDH patients. Moreover, immunohistochemical staining showed a significant increase in the proportion of NOX2‐positive cells compared to the Control group in the femoral head of rats. In vitro, Dex significantly inhibited the viability of osteocyte cells and induced apoptosis. After Dex treatment, the intracellular ROS level increased. However, Dex treatment did not alter the expression of NOX proteins in vitro. Additionally, NAC and DPI inhibited the generation of intracellular ROS and partially alleviated osteocyte apoptosis in vivo and in vitro.

**Conclusion:**

This study demonstrates that GC promotes apoptosis of osteocyte cells through ROS‐induced oxidative stress. Furthermore, we found that the increased expression of NOXs induced by GC serves as an important source of ROS generation.

## Graphical abstract

Glucocorticoid (GC) overuse is associated with steroid‐induced osteonecrosis of the femoral head (SINFH). In the development of SINFH, Dex induced apoptosis in osteocytes through ROS‐mediated oxidative stress. The Dex‐induced NOX is an important source of ROS production.

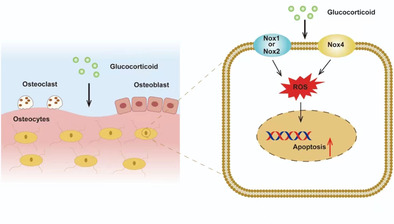

## Introduction

Oxidative stress is recognized by significant levels of reactive oxygen species (ROS) that impair intracellular reduction–oxidation equilibrium.^1^ The elevated bioavailability of ROS adversely affects cellular macromolecules, such as RNA, DNA, proteins, lipids, and carbohydrates, and leads to cellular damage and death.^2^ Oxidative stress has been shown to be involved in a variety of diseases, including hypertension, respiratory diseases, Parkinson's disease, immune surveillance, and bone remodeling disorders.^3^, ^4^ ROS is the main type of free radical involved in the destruction of bone remodeling.^5^ ROS inhibits the expression of Runt‐related transcription factor‐2 (Runx2) and osterix, reducing osteogenic activity, while promoting the expression of osteoclast markers such as C‐Fos, Nuclear Factor of Activated T cells‐1 (NFATc1), and Tartrate Resistant Acid Phosphatase (TRAP).^6^, ^7^ ROS may enhance the response of osteoclast precursors to Receptor Activator of NF‐κB Ligand (RANKL), and induce the production of additional osteoclastic factors, such as IL‐1, IL‐6, and IL‐7.^8^, ^9^ Accumulating evidence has shown that ROS‐induced oxidative stress is closely associated with the pathophysiology of steroid‐induced femoral head osteonecrosis (SINFH).^10^, ^11^, ^12^

Osteonecrosis of the femoral head (ONFH) is an irreversible, progressive disorder of the hip that eventually leads to hip dysfunction and collapse.^13^ In the past decades, glucocorticoids (GCs) have been widely used in spinal trauma, autoimmune diseases and hematopoietic disorders as a result of their powerful anti‐inflammatory, immunosuppressive, and metabolism‐modulating effects.^14^, ^15^, ^16^ In addition, GCs therapy is recommended for patients with severe COVID‐19.^17^ Although many treatments have been adopted to treat SINFH, most have failed to exhibit adequate clinical results, particularly in young patients, and artificial total hip arthroplasty has become the final option for the treatment of end‐stage SINFH.^18^ Since there is no effective treatment for young individuals with SINFH, it is vital to comprehend its pathophysiology and develop therapies that lessen or eradicate ONFH. GCs increase intracellular levels of ROS, inhibiting osteogenic differentiation while promoting adipogenesis and osteoclast differentiation, ultimately leading to an imbalance in bone formation and bone resorption.^12^, ^19^, ^20^, ^21^

Investigations have revealed that osteocyte apoptosis plays a critical role in SINFH.^22^, ^23^, ^24^ Osteocytes, comprising 90% to 95% among all bone cells, are widely distributed and maintain bone homeostasis and biomechanical performance through communicating with osteoblasts and osteoclasts.^25^, ^26^ Osteocytes are versatile cells with several essential regulatory functions in skeletal and mineral homeostasis. Osteocytes apoptosis is the earliest form of bone cell death in SINFH patients, which causes a significant decrease in osteocyte viability in the femoral head.^23^, ^27^ GCs‐induced osteocyte apoptosis is a cumulative, irreparable defect that induces cytoskeletal rearrangements by shortening the dendritic processes of osteocytes and decreasing the expression of the gap junction protein connexin 43, thereby initiating an inevitable series of events leading to joint collapse.^28^, ^29^ However, the specific mechanism of GCs‐induced osteocyte apoptosis remains unclear.

NADPH oxidase (NOX) is an important producer of ROS production.^30^ Numerous studies have shown that NOX proteins are closely related to bone homeostasis and bone remodeling.^31^, ^32^ Yang et al.^19^ found that GCs increased the expression levels of NOX1, NOX2, NOX4, and ROS in BMSCs and inhibited the activity and osteogenic differentiation of MSCs, while diphenyleneiodonium chloride (DPI) reduced the intracellular ROS levels. In a previous experiment by our research group,^33^ environments with a high level of GCs could be established in vitro using dexamethasone (Dex). We observed that the upregulation of intracellular NOX1, NOX2, and NOX4 expression was accompanied with the accumulation of ROS and apoptosis of osteoblast in a dose‐dependent manner. However, N‐acetyl‐L‐cysteine (NAC), DPI, and siRNA‐NOX reversed this effect. Thus, our study demonstrated that NOXs were an important source of ROS production in a cellular model of SINFH and the upregulated expression of NOXs were a key pathological basis for apoptosis induced by oxidative stress in osteoblasts.

However, the role of Dex‐induced oxidative stress and NOXs in osteocyte apoptosis have been little studied in SINFH. The purposes of the study were to: (i) investigate whether oxidative stress injury plays a role in Dex‐mediated apoptosis in osteocytes; (ii) explore whether NOXs are an important source of ROS production in osteocytes.

## Materials and Methods

### Reagents

Cell culture medium and other products were obtained from GIBCO (Rockville, MD). NAC and DPI were obtained from Sigma–Aldrich (St. Louis, MO, A0150000) and Santa Cruz Biotech (Santa Cruz, CA, CAS: 1483‐72‐3), respectively. Primary antibodies against NOX1 (PA5‐79752), NOX2 (PA5‐72435), and NOX4 (MA5‐32090) were purchased from Invitrogen (California, US). 8‐OHdG and TUNEL were purchased from Abcam (Cambridge, UK, ab62623) and Beyotime (Jiangsu, China, C1090), respectively.

### Harvesting and Processing of Human Femoral Head Specimens

From January 2021 to January 2022, patients diagnosed with SINFH or developmental dysplasia of the hip (DDH) who underwent total hip replacement at General Hospital of Tianjin Medical University were recruited. Patients with SINFH were classified according to the ARCO staging system, while patients with DDH were staged according to the Crowe classification. Finally, 10 patients with SINFH in ARCO stage IV and 10 patients with DDH in Crowe classification stage III or IV were recruited for the study.

During the operation, the femoral heads were collected after the femoral necks were removed. The femoral head samples were cut into coronal sections using an electric bone saw, and then the sections were modified into 1.5 cm× 1.5 cm× 1 cm specimens using bone rongeur. After the samples were repeatedly rinsed with normal saline, they were fixed in 10% formalin for 72 h and then decalcified in a solution of 10% EDTA at room temperature on a shaker for 6–8 weeks. The decalcification solution was replaced every 2 days. The decalcified tissue was embedded and cut into 6 μm sections for subsequent histological examination.

### Animal Studies

All experimental procedures were executed under the Care and Use of Laboratory Animals guidelines, which were approved by Tianjin Medical University's Animal Ethics Committee (Ethics approval number IRB2021‐KY‐138). The Tianjin Medical University Experimental Animal Center provided 60 male Sprague–Dawley rats that were 12 weeks old and weighed between 380 and 420 g. GCs‐induced femoral head necrosis was established as follows: Dex (21 mg/kg per day) was injected intramuscularly daily for 4 weeks. All rats were randomly divided into six groups (*n* = 10); saline only (control group); (2) Dex only (model group); (3) model group rats with NAC treatment (150 mg/kg per day, i. p. injection); (4) model group rats with DPI treatment (1 mg/kg per day, subcutaneous, injection); (5) NAC only (150 mg/kg per day, intraperitoneal, injection); (6) DPI only (1 mg/kg per day, subcutaneous injection). All in vivo MR imaging was performed at 9.3T MRI (Bruker, German) after 4 weeks.

### Histological Examination

After decalcification in 10% EDTA for 4 weeks, all specimens were preserved in 4% paraformaldehyde, embedded in paraffin, coronally sectioned (5 μm thick), and mounted on slides. After hematoxylin and eosin (HE) staining, sections were sealed with neutral resin and observed under a Carl Zeiss microscope (Germany).

### Immunofluorescence Staining

TUNEL staining could detect the breakage of nuclear DNA during apoptosis, while normal or proliferating cells have little to no DNA breakage. the TUNEL test is suitable for in situ detection of apoptosis in tissue and cell samples. 8‐OhdG (8‐hydroxydeoxyguanosine), an oxidative adduct generated by hydroxyl radicals and superoxide anions that would directly attack the 8th carbon atom of the guanine base in the DNA molecule, is an important marker for the evaluation of oxidative stress damage. The paraffin‐embedded sections were dewaxed and rehydrated after drying at 60 °C for 90 min. First, sections were incubated in Proteinase K and TUNEL working solutions for 30 min at room temperature, respectively. After washing three time with PBS (5 min each wash), sections were incubated in 8‐OHdG working solution (1:200 dilution) for 120 min. Five randomly selected fields per section and three sections per rat were analyzed to figure out the percentage of positive cells per field.

### Immunohistochemical Staining

For immunohistochemical staining, tissue sections were incubated overnight at 4°C with primary antibodies against NOX1 (1:200), NOX2 (1:200), and NOX4 (1:400). The sections were incubated with goat anti‐mouse secondary antibody conjugated with horseradish peroxidase (HRP). Finally, DAB staining was performed to visualize the signal of the secondary antibody, followed by hematoxylin counterstaining of the nucleus. Five randomly selected fields per section and three sections per rat were analyzed to figure out the percentage of positive cells per field.

### Cell Culture

MLO‐Y4 cells were obtained from Procell (Wuhan, China) and cultured at 37°C in a 5% CO_2_ environment in Dulbecco's modified Eagle's medium (DMEM) supplemented with 10% heat inactivated foetal bovine serum (FBS) and 1% penicillin–streptomycin. To establish the high‐dose Dex environment, MLO‐Y4 cells were treated with 1 μM Dex for 24 h as previously reported.^24^, ^34^ In certain trials, cells were pretreated for 30 min with NAC (1, 2.5, 5, 7.5, or 10 mm) or DPI (1, 10, 15, or 25 nm) prior to Dex stimulation.

### Cell Viability Assay

The cell proliferation was measured to use a cell counting kit‐8 (CCK‐8) assay. Initially, we identified the appropriate DPI and NAC concentrations for cell growth. In 96‐well plates, 5 × 10^3^ MLO‐Y4 cells were seeded per well with NAC (5 mM) or DPI (1 nM), and 10 μL of CCK‐8 solution was added to each well for a 4‐h incubation period. Second, to identify cell proliferation, the cells were separated into groups for different interventions, including control, Dex, Dex + DPI, Dex + NAC, DPI, and NAC. A microtiter‐plate reader was used to measure absorbance at 450 nm.

### ROS Measurement

5 × 10^4^ cells were seeded into six‐well plates, and different interventions were performed when the cells archived 75% confluence. MLO‐Y4 cells were incubated with DCFH‐DA at room temperature for 15 min. After washing three times, ROS were detected by flow cytometry (BD Biosciences).

### TUNEL Assay

For apoptosis investigation, 1 × 10^4^ cells were cultured into 12‐well plates for 24 h before receiving various treatments. After being washed with PBS and fixed with 4% paraformaldehyde, the cells were permeabilized with 0.3% Triton X‐100 and incubated at room temperature for 30 min. MLO‐Y4 cells were treated with 100 μL of TUNEL working solution and incubated at room temperature for 60 min. In each experiment, cells in at least five random fields were counted, and three independent experiments were conducted.

### Western Blotting

We obtained MLO‐Y4 cells and used lysis buffer to lyse them (Solarbio Biotech). Proteins were extracted by SDS‐PAGE (sodium dodecyl sulfate polyacrylamide gel electrophoresis), and then they were transferred to PVDF membranes. After blocking with fat‐free powdered milk, the membranes were incubated overnight at 4°C with antibodies against NOX1 (1:1000), NOX2 (1:1000), NOX4 (1:2000), and GADPH (1:1000, Invitrogen) and then for 120 min at room temperature with secondary antibodies. Immunoreactive products were detected using chemiluminescence (Pierce Biotechnology) and quantified using Quantity One software.

### Statistical Analysis

Experimental values were statistically analyzed using SPSS 23.0 (IBM SPSS Statistics for Windows, version 23.0, Armonk, NY, USA). Different groups were executed by one‐way analysis of variance (ANOVA). Results of *p* < 0.05 were considered to be statistically significant.

## Results

### Histomorphologic Analysis of Patients with DDH and SINFH

**FIGURE 1:**
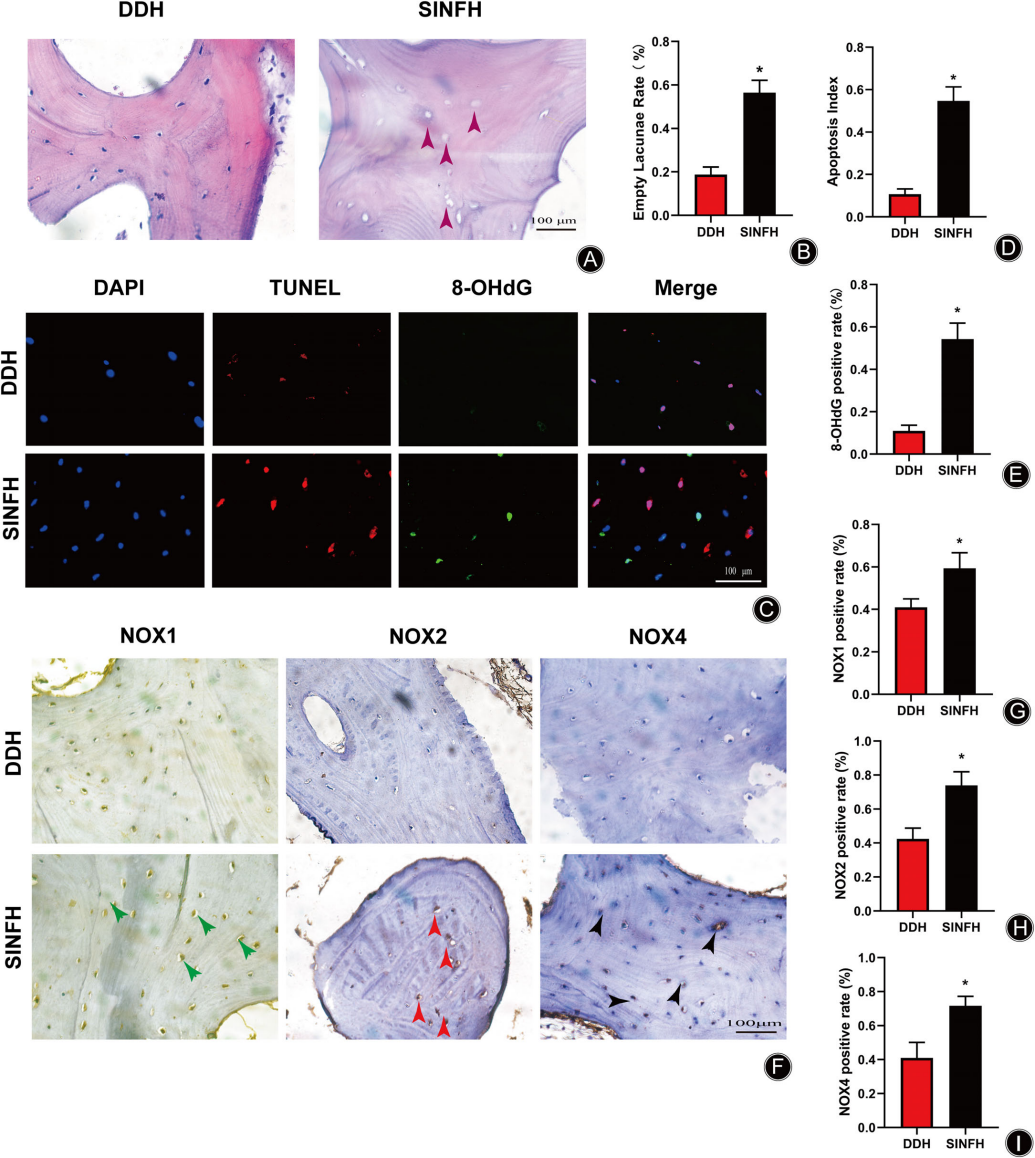
NOX is the source of GCs‐mediated ROS production in SINFH patients. (A, B) Representative HE staining images and quantitative analysis of the empty lacuna rate in the DDH and SINFH Group. (C–E) Representative immunofluorescence images and quantitative analysis of the percentage of TUNEL and 8‐OHdG in DDH and SINFH Group. (F–I) Representative immunohistochemical images of NOX proteins and quantitative analyses of the percentages of NOX1, NOX2, and NOX4‐positive cells. Purple arrow indicates empty lacuna, green arrow indicates NOX1 positive cell, red arrow indicates NOX2 positive cell, and black arrow indicates NOX4 positive cell. (*n* = 10 per group, Data are shown as the mean ± SD, *p < 0.05 vs. DDH group, scale bar = 100 μm).

DDH refers to the abnormal shape and relative position of the proximal femur and acetabulum caused by various factors in the development process. First, HE staining of decalcified bone sections was performed to investigate the histomorphologic differences between DDH and SINFH patients. In Figure 1A, HE staining showed the histopathological appearance of the femoral head in patients with DDH and SINFH. Compared with DDH group, HE staining showed a large number of empty lacunae in the subchondral bone trabeculae of SINFH patients. In addition, the formation of microfractures can be observed in the bone trabecula. However, microfractures of bone trabecula and empty lacunae were rarely found in DDH group (Figure 1B).

### Oxidative Injury and Osteocyte Apoptosis Were Involved in SINFH Patients

In order to further investigate the mechanism of GCs effect on osteocytes, we conducted the detection of osteocyte apoptosis and markers related to oxidative stress injury. 8‐hydroxy‐2‐deoxyguanosine (8‐OHdG), an oxidative adduct forming by ROS attacking DNA molecules, has become the most prevalent indicator of oxidative DNA damage. TUNEL and 8‐OHdG staining of the femoral head section were demonstrated in Figure 1C. Compared with DDH group, osteocyte apoptosis and 8‐OHdG production were significantly increased (Figure 1D,E). Therefore, we supposed that excessive useage of GCs may lead to osteocyte apoptosis by promoting oxidative stress injury of osteocytes in SINFH patients.

### GCs Promoted NOXs Production in the Femoral Head of SINFH Patients

NOXs are an important source of intracellular ROS. Subsequently, in order to verify whether GCs lead oxidative stress damage by promoting the level of NOXs, we conducted immunohistochemical staining of bone tissue sections. The immunohistochemical appearance of bone sections from patients with DDH and SINFH were displayed in Figure 1F. The levels of NOX1, NOX2, and NOX4 in the femoral head were significantly increased in SINFH patients when compared with the DDH group (Figure 1G–I). These results suggested that GCs promoted NOX production in the femoral head of patients with SINFH.

### Dex Induced Osteonecrosis in SINFH Rats

**FIGURE 2:**
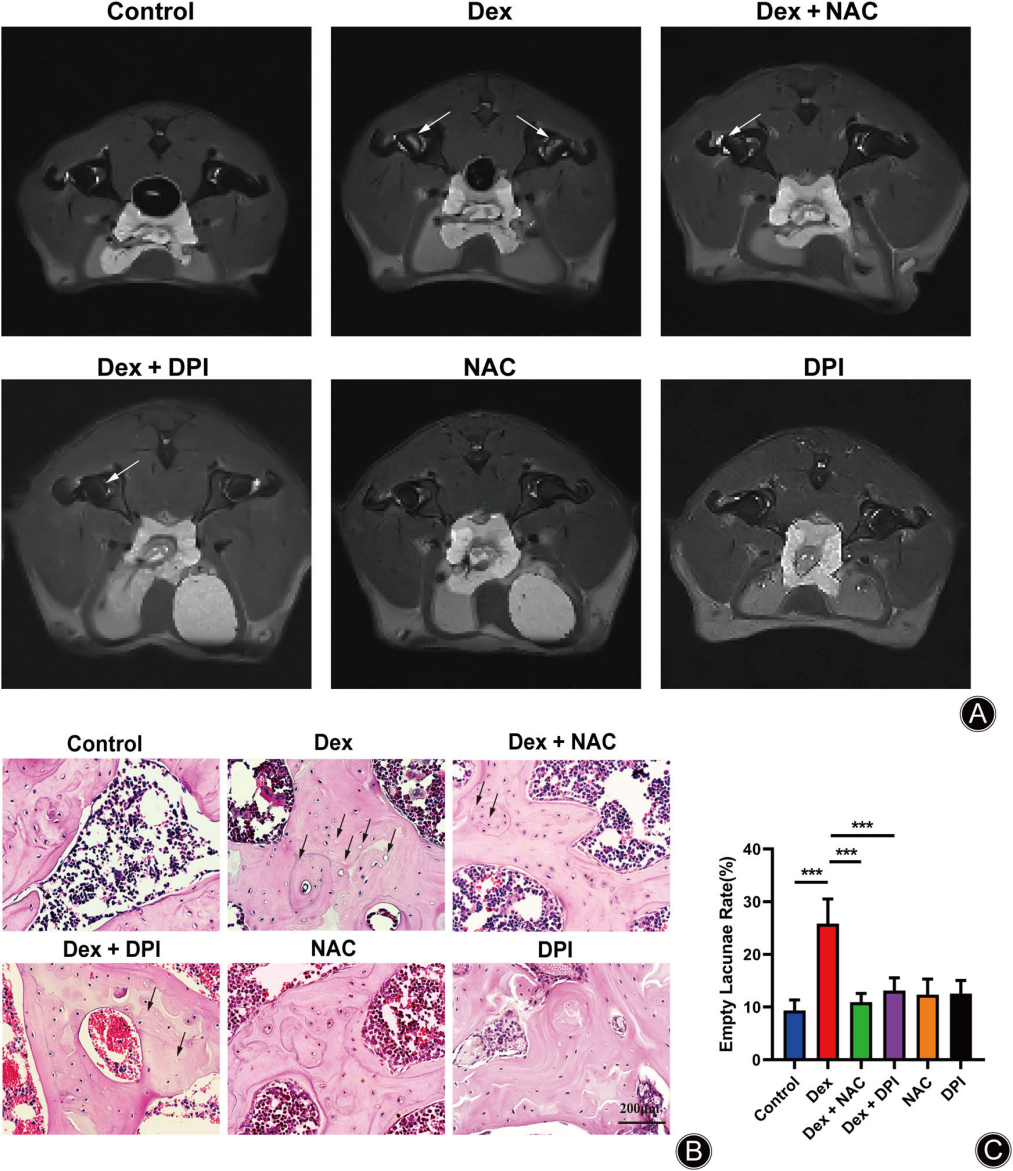
Establishment of SINFH in rats. (A) Representative MRI T2W1 images of rats in each group. The white arrows represent areas of edema and necrosis of the femoral head bone tissue. The black arrows represent the empty lacunae. (B) Representative images HE staining of rats from each group. (C) Quantitative analysis of the empty lacuna rate in each treatment group. Data are represented as the mean ± SD from three independent biological replicates. (***p < 0.001 vs. control group. HE, hematoxylin and eosin; MRI, magnetic resonance imaging; T2WI, T2‐ weighted MRI images. Scale bar: 200 μm).

We used micro‐MRI to scan bilateral hip joints to assess the effect of Dex on ONFH in rats. As shown in Figure 2A, the subchondral bone area in the Dex group displayed a clear high‐intensity signal in T2W1 compared with the normal group, which represented oedema and necrosis of the bone tissue in the femoral head. In contrast, the T2W1 signal from the subchondral bone in the NAC + Dex group and DPI + Dex group were significantly weakened and close to the surrounding bone tissue. Histomorphological features were observed by HE staining in rats, which confirmed the occurrence of SINFH. In the Dex group (Figure 2B), the trabeculae of the subchondral bone region showed the accumulation of pyknotic nuclei and empty lacunae and fibrous tissue in the medullary cavity, which were all features of severe osteonecrosis. Interestingly, in the NAC + Dex and DPI + Dex groups, there was little pyknosis and empty lacunae in the trabecular bone, which indicated that osteonecrosis was restrained (Figure 2C).

### Dex Induced Apoptosis in SINFH Rats via Oxidative Stress

**FIGURE 3:**
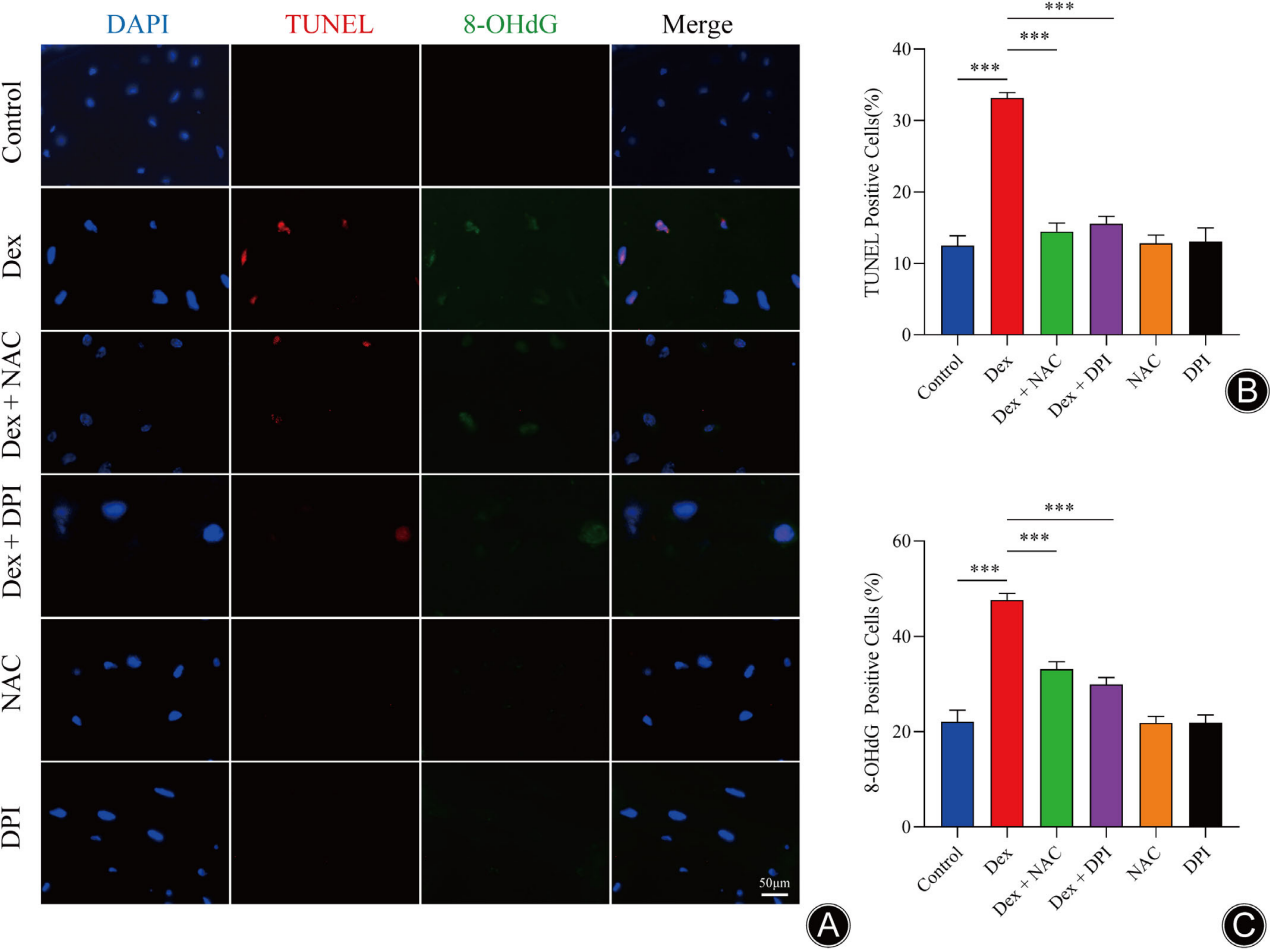
Osteocyte apoptosis induced by oxidative injury were involved in SINFH in rats. (A) Representative immunofluorescence images of TUNEL‐positive cells (red) and 8‐OHdG‐positive cells (green) in each group. (B) Quantitative analysis of the percentage of TUNEL‐positive cells in each group. (C) Quantitative analysis of the percentage of 8‐OHdG‐positive cells in each group. (*n* = 3 per group, data are shown as the mean ± SD, ***p < 0.001 vs. Dex group, scale bar = 50 μm;).

We performed TUNEL and 8‐OHdG staining to evaluate apoptosis and oxidative stress in order to better comprehend how Dex affected apoptosis in rats (Figure 3A). Figure 3B,C demonstrated that the amount of TUNEL‐positive cells and 8‐OHdG‐positive cells were considerably greater in the Dex group than in the Control group. These results suggested that GCs mediated oxidative stress and oxidative DNA damage, ultimately leading to apoptosis in SINFH rats.

### NOX Subtype Was Involved in the Progression of SINFH

**FIGURE 4:**
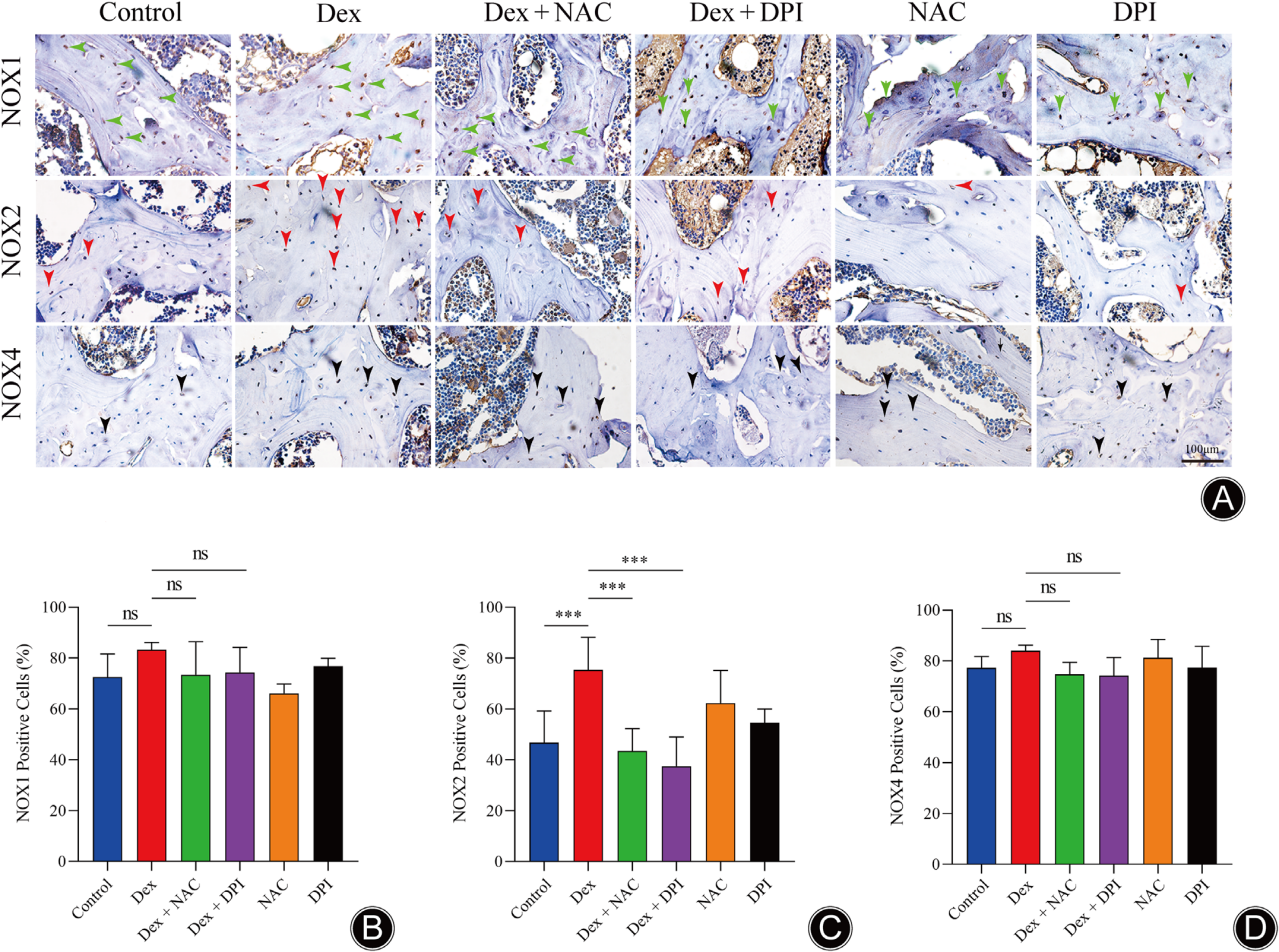
Representative immunohistochemical images of NOX proteins in SINFH in rats. (A) Representative immunohistochemical staining images of NOX1, NOX2, and NOX4. (B–D) Quantitative analyses of the percentages of NOX1, NOX2, and NOX4‐positive cells. Green arrow indicates NOX1 positive cell, red arrow indicates NOX2 positive cell, and black arrow indicates NOX4 positive cell. (*n* = 3 per group, Data are shown as the mean ± SD, ***p < 0.001 vs. Dex group, ns, no significance, scale bar = 100 μm).

In our previous in vitro studies, we discovered that NOX1, NOX2, and NOX4 isoforms triggered the generation of ROS following Dex treatment and were involved in the apoptosis of osteoblast. Consequently, we further explored the effect of GCs on NOX expression in a rat model of SINFH. The protein expression levels of NOX1, NOX2, and NOX4 were detected by immunohistochemical (IHC) methods. As shown in Figure 4A,C, NOX2 expression was significantly higher in the Dex group, while NOX1 (Figure 4B) and NOX4 (Figure 4D) expression shown no significant changes compared with the normal group.

### Dex Inhibited MLO‐Y4 Viability and Induced Apoptosis of MLO‐Y4 Cells

**FIGURE 5:**
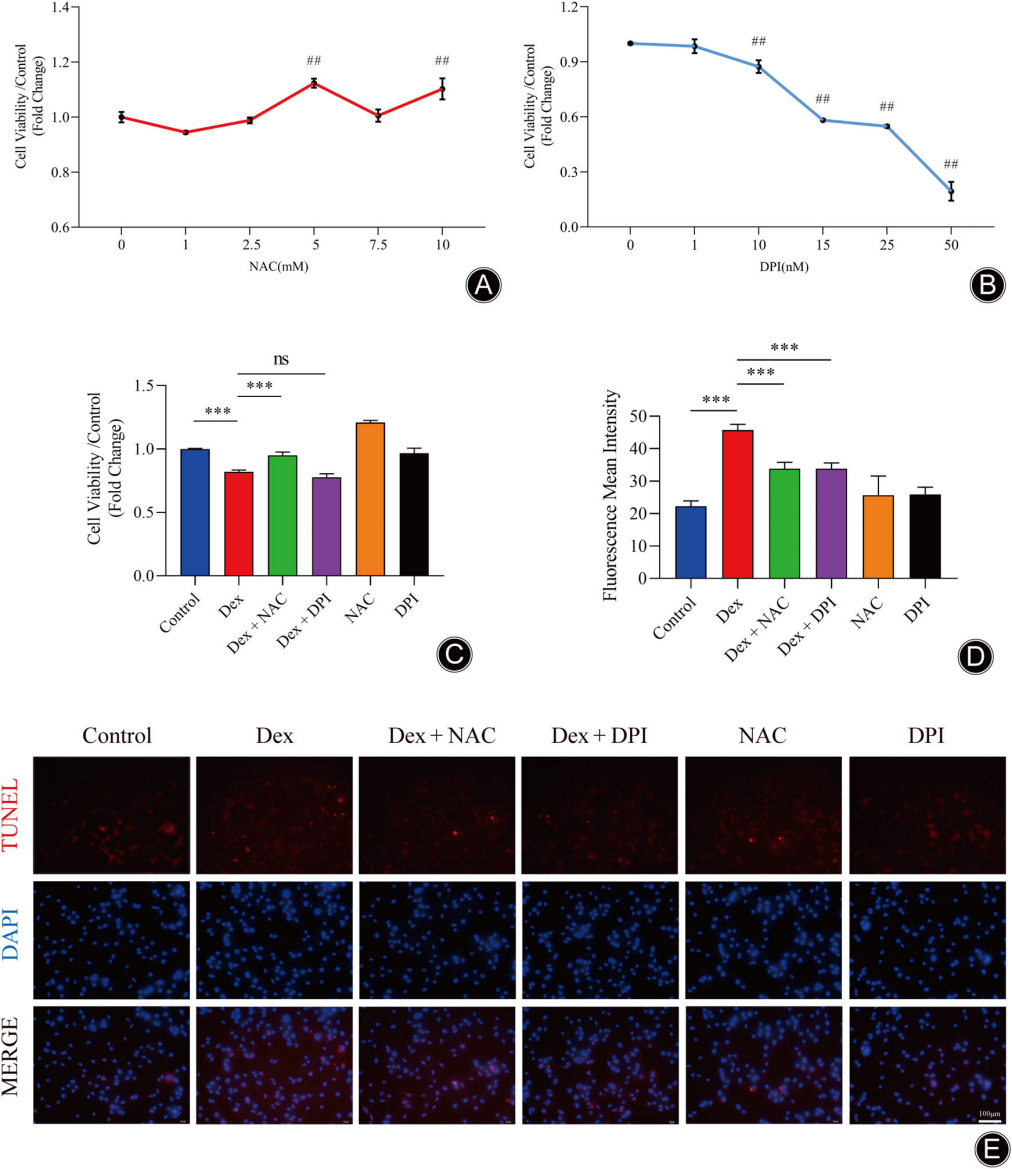
Dex inhibited MLO‐Y4 cell viability and mediated apoptosis. (A) Relative MLO‐Y4 cell viability after 24 h of treatment of MLO‐Y4 cells with different concentrations of NAC. (B) Relative MLO‐Y4 cell viability after 24 h of treatment with different concentrations of DPI. (C) Relative viability of each group of MLO‐Y4 cells after 24 h of treatment with different interventions. (D) Mean fluorescence intensity of MLO‐Y4 cells apoptosis. (E) TUNEL fluorescence staining to detect apoptosis in MLO‐Y4 cells after 24 h of Dex treatment. All data are expressed as the mean ± SD. (*n* = 3, ^##^ *p* < 0.01 vs. control group, *p < 0.05, **p < 0.01, ***p < 0.001 vs. Dex group).

In this study, the viability of MLO‐Y4 cells was inhibited by Dex, leading to the induction of apoptosis. To evaluate Dex‐induced apoptosis, MLO‐Y4 cells were treated with varying concentrations of NAC (1, 2.5, 5, 7.5, 10 mM). Upon treatment with 1 or 2.5 mM of NAC, the growth of MLO‐Y4 cells was notably impaired. However, the use of 5, 7.5, and 10 mM NAC resulted in similar cell proliferation outcomes. Consequently, 5 mM NAC was selected for subsequent experiments (Figure 5A). Then, we applied different concentrations of DPI (1, 10, 15, 25 nM) to treat MLO‐Y4 cells and found that 1 nM DPI had no significant effect on cell viability, while 10, 15, 25 nM significantly inhibited the viability of MLO‐Y4 cells. Hence, we chose 1 nM DPI for the subsequent experiments (Figure 5B). Regarding the concentration of Dex, we chose 1 μM for the experiments based on previous studies.^35^ We applied a CCK‐8 assay and TUNEL immunofluorescence staining to determine the effect of Dex on MLO‐Y4 cell viability and apoptosis, respectively. As shown in Figure 5C, compared with the normal control group, Dex treating MLO‐Y4cells with Dex the experimental group significantly inhibited cell viability after 24 h. Furthermore, TUNEL staining showed that the mean fluorescence intensity of MLO‐Y4 cells increased significantly after 24 h of Dex treatment compared with the normal control group. However, the fluorescence intensity decreased significantly in the Dex + NAC, and Dex + DPI groups (Figure 5D,E). These results suggested that Dex had the potential to induce apoptosis of MLO‐Y4 cells while NAC and DPI could reverse the negative Dex‐mediated effects.

### Dex Enhanced Intracellular ROS Levels in MLO‐Y4 Cells

**FIGURE 6:**
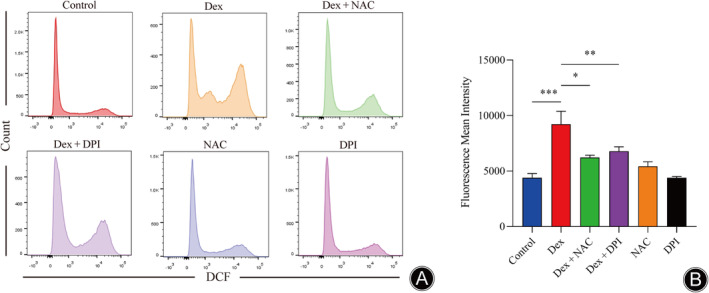
Dex induced the generation of ROS in MLO‐Y4 cells. (A) Changes in ROS levels in MLO‐Y4 cells after 24 h of treatment with Dex were measured using a DCFH‐DA Assay Kit. (B) Quantitative analysis of the mean fluorescence intensity of ROS in MLO‐Y4 cells. (*n* = 3, *p < 0.05, **p < 0.01, ***p < 0.001 vs. Dex group).

To further investigate whether Dex‐induced apoptosis was associated with increased ROS production, we applied flow cytometry and DCFH‐DA staining to detect the ROS levels in MLO‐Y4 cells after Dex treatment. As shown in Figure 6A,B, the mean fluorescence intensity in MLO‐Y4 cells was significantly increased after Dex treatment, which indicated that Dex increased the ROS level in MLO‐Y4 cells. However, NAC and DPI intervention significantly reduced the intracellular ROS levels.

### Dex Did Not Alter the Protein Level of Cellular NOX in MLO‐Y4 Cells in vitro

**FIGURE 7:**
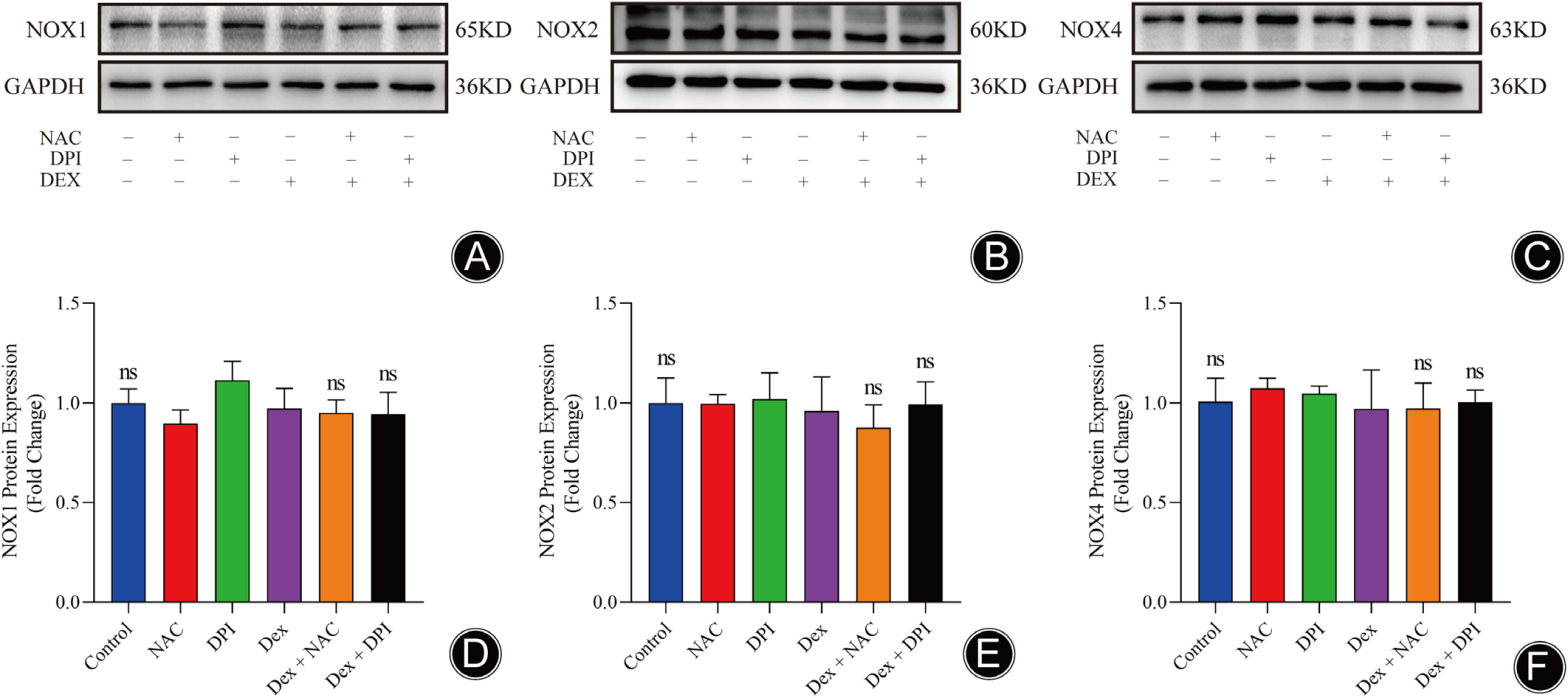
Protein expression levels of NOX1, NOX2, and NOX4 after Dex treatment in the differentially treated MLO‐Y4 cells. (A–C) Representative immunoblots of NOX1, NOX2, NOX4. (D–F) Quantification analysis of NOX1, NOX2, and NOX4 expression. (*n* = 3, Data are shown as the mean ± SD, ns, no significance).

Previous research has demonstrated that NOXs, including NOX1, NOX2, and NOX4, are indispensable for the Dex‐induced generation of ROS in osteoblasts.^33^, ^36^ As a result, we investigated the effect of Dex on NOXs expression to explore whether NAC and DPI can reduce Dex‐induced ROS generation by modulating these enzymes. As shown in Figure 7A–F, the protein expression levels of Nox1, Nox2, and Nox4 were not significantly changed after Dex treatment.

## Discussion

Steroid‐induced osteonecrosis of the femoral head has emerged as a prevalent condition associated with a substantial disability rate.^18^ In recent years, SINFH diagnosis and treatment have achieved significant progress. However, the underlying pathogenesis remains unclear. Among the various hypotheses, osteocyte apoptosis is one of the most studied pathogenic mechanisms. In the present study, we confirmed that GCs induced osteocytes apoptosis through ROS‐mediated oxidative stress. Furthermore, in patients and in vivo experiments showed that the GCs‐induced NOXs is an important source of ROS production. In addition, the antioxidants DPI and NAC reduced ROS generation in MLO‐Y4 cells after Dex treatment, enhanced cell viability, and alleviated SINFH.

### The Establishment of Rats SINFH Model

Well‐established animal models play an extremely crucial role in the study of the mechanisms of GCs‐induced ONFH. Rats have been identified as a low‐cost animal for developing GCs‐induced ONFH model. However, no generally acknowledged induction model exists. Zheng et al.^37^ successfully induced ONFH in rats by injecting LPS and MP, however, the mortality rate exceeded 15%. Therefore, we adjusted the modeling protocol to decrease the mortality rate. Fortunately, no rats (0/10) died, and 75% (7/10) of the rats in the model group presented with typical ONFH features. These results indicate that our rat model of ONFH induced by Dex may be an appropriate preclinical animal model.

### GC‐Induced Apoptosis of Osteocytes Plays a Pivotal Role in the Progression of SINFH

Glucocorticoid overdose has become a major pathogenetic factor in nontraumatic femoral head necrosis, and GCs‐induced apoptosis of osteocytes plays a pivotal role in the progression of SINFH.^18^ Osteocytes are in charge of mechanosensory and mechanotransduction in bone tissue. By sensing fluid flow shear stress, osteocytes promote the release of NO, ATP, and prostaglandins, induce the opening of connexin 43 hemichannels, and enhance gap junction functions to inhibit osteocyte apoptosis.^38^ Osteocytes convert mechanical signals into biological signals by regulating cytokines.^39^ These signals are transmitted from deeply buried osteocytes to osteoblasts and osteoclasts on the bone surface while coordinating the process of bone formation and resorption through the lacunocanalicular system. Previous studies have shown that the adverse effects of Dex on osteocytes are mainly due to the inhibition of osteocyte viability and proliferation and the promotion of apoptosis.^27^, ^40^ In our study, we found that Dex significantly promoted apoptosis in MLO‐Y4 cells, which was accompanied by a decrease in cell viability. These results are consistent with earlier research.^24^, ^27^

### Glucocorticoids Induce Apoptosis in Osteocytes by Promoting ROS Accumulation

Numerous studies have shown that ROS‐induced apoptosis is involved in many disease processes.^2^, ^3^ Excessive ROS contribute to increased levels of intracellular ROS, leading to apoptosis by increasing intracellular Ca^**2+**^ levels and reducing GPx activity, causing protein, lipid and DNA damage and activating mitochondria‐mediated apoptotic pathways.^1^, ^2^, ^3^ In the present study, we found that Dex treatment increased ROS levels in MLO‐Y4 cells, while coculture with NAC significantly decreased the intracellular ROS levels and reversed Dex‐mediated apoptosis. These findings provide additional evidence that the proapoptotic effect of Dex on osteocytes is mediated by increased ROS production. This finding is consistent with that of Wang et al.^23^ who showed that increased levels of ROS caused apoptosis and that the application of ROS‐scavenging inhibitors prevented apoptosis.

### NOXs‐Mediated ROS Production May Play a Key Role in the Progression of SINFH

NADPH oxidase is an oxidase and important intracellular ROS producer that plays a key role in oxidant responses.^3^, ^30^ The diverse Nox family members notably differ in their activation methods, tissue localisation, and subcellular localization, as well as in their expression patterns, which are cell‐specific.^1^ According to earlier research, osteoblasts express NOX1, NOX2, and NOX4, but not NOX3, which is consistent with other investigations.^41^ The contribution of NOX1 to bone conversion is unclear and remains controversial. NOX2 and NOX4 have similar roles in bone reconstruction, as both contribute to osteoclastogenesis and bone homeostasis. In our current experiments, we observed that NOX1, NOX2, and NOX4 significantly contribute to the oxidative damage of osteocytes in patients with SINFH. Consistent with previous findings, our results validate the activation of NOX isoform family proteins and the promotion of ROS production in osteocytes by GCs. In our rat models of SINFH, GCs were observed to upregulate the protein expression of NOX2, whereas NOX1 and NOX4 showed no significant alterations. The disparities in results between SINFH patients and rats can potentially be attributed to interspecies variations. Importantly, the application of DPI and NAC effectively reduced the levels of oxidative stress and osteocyte apoptosis. These findings indicate that NOX serves as a significant contributor to ROS production, and the use of antioxidants could prove to be an effective therapeutic approach in managing SINFH. Regrettably, the protein expression levels of NOX1, NOX2, and NOX4 did not exhibit significant alterations in the in vitro experiments when compared to the control group. Several possibilities can be speculated for this result: firstly, NOX enzymes are multisubunit combinatorial enzymes whose activation relies on the migration and assembly of subunits from the cytoplasm to the plasma membrane. It is possible that the mechanism by which Dex promotes the activation of NOX proteins involves facilitating the assembly of NOX cytoplasmic subunits and plasma membrane subunits, thereby enhancing the activity of NOX proteins without affecting their expression. Consequently, no notable alterations were observed in the protein content of NOX enzymes following Dex treatment in MLO‐Y4 cells during the Western Blot experiments. Furthermore, Dex might induce the expression of NOX proteins by modifying fluid shear stress within the lacunar–canalicular system of osteocytes or by promoting the influx of intracellular calcium.^42^ Alternatively, Dex could impact NOX protein expression in osteocytes by modulating intercellular signaling. Cells cultured ex vivo are isolated from the organism's internal environment, leading to the loss of contact and response to other cells or signaling molecules. Consequently, in vitro experiments do not accurately reflect the in vivo environment, often resulting in inconsistencies between in vivo and in vitro experimental outcomes. Future investigations will involve designing more rigorous experiments to delve deeper into the mechanisms underlying Dex‐mediated regulation of NOX protein expression.

### Limitations

However, one potential limitation of our study is the lack of testing the impact of Dex on the protein expression of NOX subunits and the measurement of intracellular NOX mRNA levels in vitro.

## Conclusion

In conclusion, we have verified that GCs induce apoptosis in osteocytes through ROS‐mediated oxidative stress. Moreover, evidence from patient studies and in vivo experiments demonstrated that GCs‐induced NOX serves as a significant contributor to ROS generation. We propose that targeting oxidative stress and suppressing NOX protein expression could serve as a therapeutic or preventive approach against SINFH.

## Conflict of Interest Statement

None of the authors has any conflict of interest to declare.

## Ethics Statement

This study was approved by the ethics committee of the Tianjin Medical University General Hospital Institutional Review Board. The animal experiment was performed in accordance with the National Institutes of Health guidelines for the use of experimental animals, and the Institutional Animal Care and Use Committee of Tianjin Medical University approved all animal protocols.

## Author Contributions

Study conception and planning: Xinglong Zhang and Huafeng Zhang. Experimental work: Xing long Zhang, Zhenhuan Yang, Ran Pang. Data analysis: Chunlei Xu, Wei Shi. Manuscript composition: Xinglong Zhang, Kai Zhang, Xinyu Liang. Conceptualization: Qian Xu. Project administration: Zhi Jun Li. Writing—reviewing and editing: Hui Li. All authors discussed the results and commented on the manuscript. Xinglong Zhang and Zhenhuan Yang contributed equally to this work.

## Funding Information

The authors acknowledge that they received no external funding in support of this research.

## Data Availability

The data used to support the findings of this study are available from the corresponding author upon request.
